# Health Tract, No. 83

**Published:** 1865

**Authors:** 


### HEALTH TRACT, No. 83.

### THE PLACELESS.

"There are fifty applicants for every vacancy, and no more will be received," was placarded on the Post-Office door on the inauguration of our new Postmaster the other day. In any large city there are a dozen applications; yes, a hundred! within half a day after the publication of any vacancy. On the incoming of a new governor or president, the "place" seekers are numbered by hundreds, thousands, and tens of thousands; and sometimes the "outside pressure" is so resistless, that the very highest officers in the government feel themselves obliged to favor persons who are strangers to them, in preference to men to whom they are under special and personal obligations, and whom they know to be fully qualified for all the duties of the station. Public men who have offices in their gift, often feel themselves compelled to bestow them on persons whom they know are not the best adapted to the position, as rewards for past political services, for present political influence, or for those conciliations of opposing parties which seem to them are indispensable to the situation of affairs. Yet opposed to these accepted applicants are men of integrity undoubted, of a refinement, of a culture, and of a once social position, which ought to guarantee success, brought to this suppliant attitude for "place" by sickness, by accident, by pecuniary revulsion, or by the perfidy of men, against which no human foresight could provide. Recently, a high name in this community, which five years ago wielded the wand of power in financial circles, was handed in for a "place" of trust and profit. Gray-headed and bald and bent, he craved the "influence" of influential men with hot tears; and after weeks and months of such debasement, and of agonizing suspenses, he failed of his object, the poor-house looking himself and helpless family full in the face. Young men and young women, within a week of this writing, have been driven into suicide, in New-York City, having vainly sought "places," until on the verge of starvation, and to escape it, took the rope and the poison. Why all this? Because they grew up without a positive occupation, without having been instructed in any handicraft. There's truth in Franklin's saying, that the "parent who brings up a son without a calling, teaches him to be a thief." Let that father then, who wishes to be assured that his son shall not languish in a penitentiary, or perish on a gallows, give that son a trade. Let the mother who desires to make it certain that the daughter she so much loves shall not pine away in some cheerless hospital, ay, some insane asylum, teach that daughter the perfect use of her needle, or better, the skillful handling of a sewing-machine; and more, how to keep a tidy house; how to prepare a comfortable meal; how to spread a well-appointed table—to do all these things with thoroughness. Such a young woman can never come to want; can never fail to find a well-paying place in this country. There are a thousand families in New-York any day, who would consider themselves "fortunate" in having such seamstresses, house-girls, nurses, and cooks, at twenty per cent higher wages than generally prevail. A good mechanic can always find work for his "victuals and clothes," with increasing wages as his fidelity and skill become known, and thus prevent that distressing sadness, that debasing cringing, that eating out all life's gladness, which wither the heart and waste away the health, until the friendly grave ends the torture.

